# Theoretical exploration of optimal metabolic flux distributions for extracellular electron transfer by *Shewanella oneidensis* MR-1

**DOI:** 10.1186/s13068-014-0118-6

**Published:** 2014-08-27

**Authors:** Longfei Mao, Wynand S Verwoerd

**Affiliations:** Centre for Advanced Computational Solutions, Department of Molecular Biosciences, Lincoln University, Ellesmere Junction Road/Springs Road, Lincoln, 7647 Canterbury Plains New Zealand

**Keywords:** MFC, Microbial fuel cell, *Shewanella oneidensis* MR-1, Bioelectricity, Flux balance analysis, Flux variability analysis, Flux minimization, FATMIN, Microbial metabolism

## Abstract

**Background:**

*Shewanella oneidensis* MR-1 is one of the model microorganisms used for extracellular electron transfer. In this study, to elucidate the capability and the relevant metabolic processes of *S. oneidensis* MR-1 involved in an electron transferring environment, we employed genome-scale modelling to model the necessary metabolic states and flux adjustments for electricity generation in the cytochrome c-based direct electron transfer (DET) mode, the NADH-linked mediated electron transfer (MET) mode and a presumable mixed mode comprising DET and flavin secretion. These are difficult to develop experimentally.

**Results:**

The results showed that the microbe had the potential to achieve current outputs of up to 2.610 A/gDW in the DET mode, 2.189 A/gDW in the MET mode and 2.197 A/gDW in the mixed mode. Compared with the DET mode, which relied on cytochrome c oxidase (EC: 1.1.1.2) to mediate the electron transfer, the MET mode was mainly dependent on two routes, catalysed by isocitrate dehydrogenase (NAD) (EC: 1.1.1.4) and NAD transhydrogenase, for the computed high current density value. In the mixed mode, whereas the cytochrome c-based DET accounted for most of the computed maximum current output value, the two flavins combined, riboflavin and FMN, played a much less important role in the probed current value.

**Conclusions:**

*Shewanella oneidensis* MR-1 has the potential to sustain a high extracellular electron transfer rate similarly to *Geobacter sulfurreducens,* but relies on different intracellular mechanisms. Various levels of electron transfer rates are achieved by different combinations of metabolic pathways. Flavins can significantly degenerate the maximum electricity generation capability of the cell and the biomass formation, and thus should be avoided in order to achieve a high coulombic efficiency.

**Electronic supplementary material:**

The online version of this article (doi:10.1186/s13068-014-0118-6) contains supplementary material, which is available to authorized users.

## Background

*Shewanella oneidensis* MR-1 is a facultative aerobic, Gram-negative bacterium capable of using various reducing exogenous extracellular acceptors such as Fe (III) to preserve energy for growth [1,2]. Due to this ability, *S. oneidensis* MR-1 has been used in microbial fuel cells (MFCs) for wastewater treatment and bioelectricity generation [3,4]. In MFCs*, S. oneidensis* can potentially produce electricity through three modes: 1) the direct electron transfer (DET) mode based on c-type cytochromes on the outer membrane of the cell and cell appendages called conductive pili (that is, nanowires), 2) self-secretion of flavins to convey electrons toward the electrode and 3) the mediated electron transfer (MET) mode, which relies on exogenous mediators (reviewed in [5–7]).

The DET route depends on the c-type cytochrome (MtrC and OmcA) in the cytoplasmic membrane interacting with the electrode and supplying electrons to the electric circuit [8]. The DET mode can also use conductive pili to transfer electrons to an electron acceptor that is located distantly from the cells. Two hypotheses were proposed for the electron conduction in nanowires of *Geobacter*: the first claims that the cytochromes mediate the electron transfer via the pilus with metallic-like conductivity, and the second suggests that the electrons are hopping between heme groups in cytochromes aligned with the pilus to reach remote electron acceptors [9]. These pili were first described for *Geobacter sulfurreducens* [10–12], and afterwards for *S. oneidensis* [12,13]. Both direct contact and nanowires are fundamentally determined by the catalytic activity of outer membrane c-type cytochromes [8]. The metabolisms of *G. sulfurreducens* and *S. oneidensis* have been studied extensively and proposed as classic models in MFC research [14,15]. Unlike the anaerobic *Geobacter*, *Shewanella* can use oxygen and thus can have wider applications.

Without a direct contact with external electron acceptors via pili, *S. oneidensis* can still reduce a remote electron acceptor by producing flavins into the surrounding medium to serve as extracellular electron shuttles [16,17]. The flavin derivatives secreted by the strain MR-1 have been specified as riboflavin and flavin mononucleotide (FMN) (or riboflavin-5’-phosphate) [18,19]. The secretion of flavins into the surrounding environment could contribute significantly to the electron transfer to electrodes, and the removal of riboflavin from biofilms reduced the rate of the electron transfer to electrodes by >70% [18]. The flavins-based electron generation was found to be closely linked to the availability of c-type cytochrome. The previous study indicated that the availability of MtrC and OmcA of the Mtr respiratory pathway determines the availability of reduced flavins [20,21]. This has raised a question regarding how the cytochrome c production rate relates to flavin and growth rates; for example, does a different level of cytochrome c abundance lead to a different reduction rate of the flavins? Furthermore, the flavin-based electron transfer mode can presumably operate simultaneously with the c-type cytochrome-based DET mode, resulting in a mixed mode involving the two types of electron transfer concurrently.

For the MET mode, the reduced form of nicotinamide adenine dinucleotide (NADH) has been suggested as the optimal intracellular electron-shuttling compound to be targeted in electricity generation [22], because NADH is a universal electron carrier connecting many different cellular processes in all living cells. Therefore, studying the current production in MFCs linked to the NADH/NAD^+^ cycle could be considered as a way to probe the maximum metabolic potential for ideal electricity generation.

In conventional MFC research, a few pathways (for example, the Mtr respiratory pathway [5,23]) can be postulated for the reduction of insoluble extracellular electron acceptors, but the complete intracellular metabolic mechanisms underlying the regeneration of redox molecules remain unclear. Because microorganisms can use different enzymes and pathways to respire, it is difficult for reductionist investigative methods (such as genomic analysis) to identify the mechanisms that are not directly involved in the observed functional phenotypes such as the extracellular electron transfer [24]. To overcome the burden encountered by the lab-based approaches, a genome-scale mathematical model that integrates information of genomics, metabolomics, and proteomics can be used to derive simple principles from the complexity of the biological system [25]. For example, recent studies have devoted efforts to develop stochastic models to investigate the electron transfer mechanism and communication occurring amongst individual *Shewanella* cells in a microbial biofilm [26–28], which signifies a need to reveal the intracellular metabolic potential of the microbe for the extracellular electron transfer.

Previous findings showed that an MFC using a pure culture of *Shewanella* with a certain level of oxygen was able to achieve higher maximum current outputs [29–31]. Therefore, for the present study, we aimed at using the modelling approach to quantitatively analyse the whole metabolism of *S. oneidensis* MR-1 under aerobic conditions, in order to identify its maximum potential for electricity generation and the pertaining metabolic mechanisms. To achieve this goal, we followed a five-step procedure. 1) Seven assumptions were made based on previous experimental findings for the modelling (see the Methods section). 2) A flux balance analysis (FBA) was performed to examine the maximum amperage output of metabolic-driven electricity generation based on *S. oneidensis* MR-1 in three operation modes: cytochrome-c linked DET mode, NADH-dependent MET mode and a mixed mode based on the availability of cytochrome c and flavins. 3) We then demonstrated theoretical trade-offs among the amperage yields, the biomass production (growth) rates, the flavin secretion rates and the cytochrome c regeneration rate. 4) The fundamental metabolic mechanisms behind the current outputs in the three modes were elucidated by flux variability analysis with target flux minimization (FATMIN). 5) Finally, a sensitivity analysis was conducted to investigate the effect of varying lactate uptake rate on the growth rate and the amperage output in the three modes, and the computed current outputs were compared and discussed.

## Results and discussion

### Impact of the redox perturbation on the biomass production in the DET and MET modes

**Figure 1:**
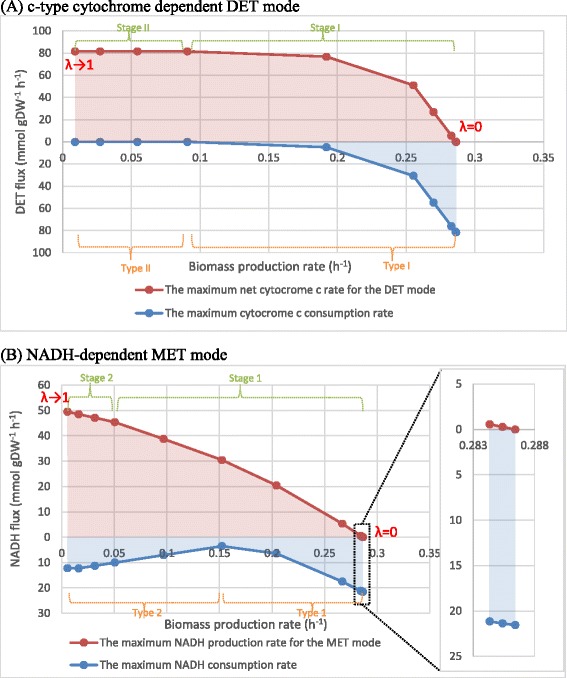
**The relationship of the biomass production and electron transfer rates.** The reducing equivalent supplying rates in the **(A)** DET and **(B)** MET modes, and the reducing equivalent consumption rate for cellular use in the **(A)** DET and **(B)** MET modes, as functions of biomass yield. The line represents the maximal electron transfer rates and biomass production rate, while any point within the pink area represents all allowable electron transfer rates and biomass production rates. The blue area represents the total reducing equivalent-consuming flux for normal cellular function. The distance between the two lines across the pink and blue areas represents the total available reducing equivalent flux in the cell at a metabolic state related to a specific biomass production rate; inset, enlargement of the boxed area. The reducing equivalent represents NADH in the MET mode, whereas it denotes an assumed product of the reaction catalysed by cytochrome c reductase for the DET mode. Stages I-II (1-2) are indicated by green braces and arrows, and Types I-II (1-2) by orange braces and arrows. The points are simulated by bi-objective optimization involving varying coefficients (λ) assigned for the growth and DET or MET maximizations. λ for DET or MET gradually increased from 0 to near 1; the two extreme points produced by the lowest λ and the highest λ are indicated in the plot. A more detailed discussion of the significance of λ is given in the Methods section. Briefly, it is a parameter that continuously adjusts the metabolic state of the cell through a range stretching from pure growth without the extraction of current (at one extreme) to a state in which all metabolic resources are taken up by supplying electric current and no growth, at the other extreme.

Figure 1 shows how production of the electron transfer (MET and DET modes) competes with biomass production for metabolic resources. The electricity generation of both the MET and DET modes drove the fluxes through the electron transfer reactions towards their maximum allowable values and corresponding biomass formation rates to their minimum values. However, in the DET mode, the electron flux sustaining the current output reached a plateau when the growth rate was about 0.1 h^-1^ (about 35% of the optimal growth 0.286 h^−-1^), whereas in the MET mode, the electron flux always increased with the decrease in the growth rate. The slope of the DET and MET outputs versus biomass growth curve as shown in Figure 1 gives a direct measure of the efficiency of the underlying pathways. A large slope means that the current production in either one of the two modes increases by a large amount for a given cost in terms of biomass production and is therefore highly efficient, and vice versa. In the discussions below, we base interpretations of reallocations of metabolic resources to pathways with different efficiencies on this evidence directly derived from the modelling results.

Starting from the unperturbed state of zero reduced cytochrome c production at the right-hand side of the plots, the relationships between varying biomass production rate and the DET rate can be divided into two stages (see Figure 1): Stage I, an increase in the DET rate not only reduced the biomass production rate, but also decreased the conversion efficiency of metabolic resource into the DET flux. This is reflected by the downward curvature associated with decreasing slopes of the lines between the data points, as the curve is followed from right to left; Stage II, at the left-side tail of the net cytochrome c production curve, a further decrease in the biomass production rate below 0.09076 h^-1^ did not improve the DET rate, forming a steady plateau at the DET output of 81.68 mmol gDW^-1^ h^-1^. Likewise, the MET production curve can also be divided into two stages: Stage 1, an increase in the MET rate was accompanied with a decrease in the biomass production rate, but the conversion efficiency of the metabolic resource into the MET flux decreased with the decline in the MET output; Stage 2, at the left-side tail of the net NADH production curve, the rise in the MET rate was linearly proportional to the drop in biomass production rate, indicating a constant efficiency. On the other hand, as a result of increasing DET or MET outputs, the relationships between the varying biomass production rate and the NADH consumption for maintenance (Figure 1) can also be classified into two types. For the DET mode, Type I denotes a rise in the cytochrome c consumption, resulting from a decline in the biomass production. Type II represents a metabolic state at which all the cytochrome c was diverted towards the electrode and further effort for improving the cytochrome c production rate did not result in any actual increase in the DET flux. For the MET mode, Type 1 was similar to Type I for the DET mode, in that the NADH consumption rate experienced a gradual drop with the decrease in the biomass production rate; Type 2 is a rise in the NADH consumption resulting from a decline in the biomass production.

Looking at the net NADH production (MET) curve, Stage 1 of the MET production curve describes the compromise between the two goals, maximizations of biomass growth and NADH production. The higher growth rates correspond to more active metabolic pathways available to cope with the NADH drain, and thus the metabolism tends to choose those pathways with higher efficiency in converting the metabolic resource originally for the biomass production to the current output. As the current output augments, which means that the conversion efficiency becomes less important than the flux capability, the metabolism has to abandon the pathways with high conversion efficiency but lower maximum capability and reallocate the metabolic resources to those with higher flux capability.

The Stage 2 behaviour sets in at extremely low growth rate values (less than about 10% of the maximum growth rates) in each of the two modes. In this stage, the reactions with lower catalytic efficiency in NADH regeneration were inactivated to leave the metabolic resources to a limited set of reactions with higher efficiency, in order to produce the extremely high current output. From an optimization point of view, the Stage 2 behaviour corresponds to the fact that the solution space was greatly reduced by the high MET rate and the remainder solutions are rigid in the selection of reactions. As a result, the decrease in the biomass production rate was linearly proportional to the increase in the MET flux.

The right part of Stage 1 of the NADH production behaviour coincides with Type 1 of the NADH consumption behaviour, which means that the increase in the MET is accompanied by a drop in the NADH consumption for cellular maintenance. This indicates that, at those production rates of biomass and NADH (corresponding to Type 1), the increase in the flux of NADH_mfc (that is, the net NADH flux diverted for current production) originates from a combination of two mechanisms: increasing the NADH production and decreasing the NADH needed for biomass production and maintenance.

The left parts of the Stage 1 and Stage 2 productions are correlated with Type 2 consumption, at lower biomass production rates. Type 2 behaviour may be contrary to intuitive expectations, since a reduction in biomass growth and an increase in NADH demand would be anticipated to reduce the NADH consumption rate. However, the increased consumption of NADH is also a way to increase the availability of NAD^+^, a building block for NADH production. Thus, Type 2 consumption of NADH is a metabolic mechanism enabling *Shewanella* to achieve higher current outputs. In addition, the increased consumption of cytosolic NADH can be considered as a metabolic strategy to reallocate the metabolic resources to those reactions that can regenerate NADH more efficiently.

Overall, compared with the base state optimized for growth, the metabolism of *Shewanella* did not increase the “ceiling” regeneration rate of the reduced cytochrome c when current was generated in the DET mode. A flux value of 81.68 mmol/gDW/h for the reduced cytochrome c production was necessary for the optimal growth rate of 0.2864 h^-1^, and a metabolic perturbation of a drain flux of cytochrome c entailed a compromise on the growth rate. This suggests that the capability of the *Shewanella* cell for regeneration of the reduced cytochrome c is inherently fixed. For the MET mode, compared with the base state optimized for growth, the metabolism of *Shewanella* had a potential to increase the NADH regeneration rates by about 130% (2.297-fold) under the highly NADH-perturbed metabolic states.

**Figure 2:**
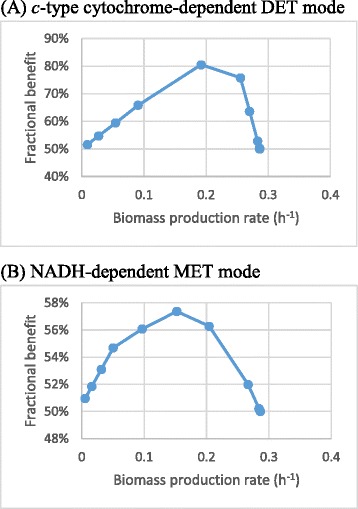
**The effect of varying biomass production on the fractional benefit values of (A) cytochrome c and (B) NADH production.** The fractional benefit *B*, plotted on the vertical axis, is a measure of success in achieving the combined goals of maximal growth rate and DET or MET flux. Maximizing one of these at a time, as at the endpoints, gives only *B* = 50%. The graphs show that relative to this, gains in DET or MET flux can more than offset losses of growth rate in the *S. oneidensis* MR-1 metabolism.

Although the discussion above indicates that the reducing equivalent production competes with the biomass growth for metabolic resources, not all the gains in reducing equivalent production are derived from the decrease in the growth rate, and some of them can result from reconfiguration of metabolism. We used a fractional benefit measure, the *B* value, to quantitatively compare the suitabilities of different combinations of multi-objectives, so as to identify the metabolic state that can most efficiently coordinate pathways for achieving the objectives. The higher the calculated *B* value achieved, the more efficiently the metabolic state can produce the metabolite of interest. A detailed explanation for the calculation of a *B* value can be found in the Methods section. Figure 2 shows the result of this measure applied to the reported simulations.

The *B* values increased from 50% at wild-type growth rates (right-hand side of the figure), to reach an apex of near 81% at a growth rate of 0.192 h^-1^, and then dropped to about 51% when the growth rate approached zero. The initial increase in the *B* values indicates that there is a rise in the metabolic output of the combined objectives - a small redox disturbance causes an adverse effect on the growth, but a further increase in the degree of the redox perturbation can improve the metabolic output of the combined two goals. The highest *B* value, about 81%, suggests that the corresponding metabolic state with a growth rate of 0.192 h^-1^ possesses the best output of the multi-objective. The subsequently declining *B* values indicate that the further, forced, increase in the DET is detrimental to biomass production. Similar to the *B* value curve of the DET mode, the *B* values calculated for the MET mode generally increased from a base value (50%) for the wild-type growth rates to reach an apex (57%), the best metabolic state for the dual objectives, and then decreased to the base value (50%), since the further improvement in reducing equivalent generation rate was at a high cost of biomass production rate. However, since the maximum *B* value could not exceed 57%, it is indicated that MET could not liberate the maximum metabolic capability of the cell for growth-coupled current production.

Notably, the highest *B* value achieved for the DET mode (81%) was much higher than that for the MET mode (about 57%). This indicates that the metabolism of *S. oneidensis* MR-1 is much more suitable for the cytochrome c-based DET mode than the NADH-linked MET mode. In other words, the metabolism favoured the DET mode more than the MET mode for higher growth rates.

**Figure 3:**
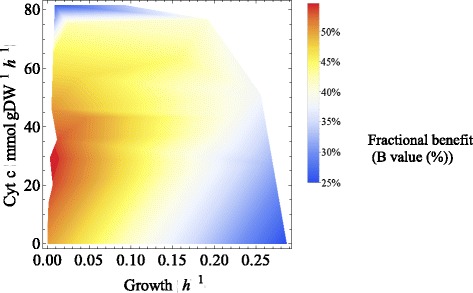
**The effect of varying biomass and cytochrome c (Cyt c) production rates on the fractional benefit value of the flavin secretion.**

Turning now to flavin-based current production, Figure 3 indicates that, to liberate the maximum capability of the flavin production, the *Shewanella* strain needs to reduce the biomass production rate to a very low level and increase the reduced cytochrome c production rate to about 30 mmol/gDW/h. It was found that the highest *B* value of about 54.6% was achievable when the growth rate was lowered to 0.003 h^-1^ and the cytochrome c production rate stayed at about 29.36 mmol/gDW/h. A further increase in cytochrome c can compromise the capability of the cell to secrete flavins.

### The effect of the flavin secretion on the output of the DET mode

**Figure 4:**
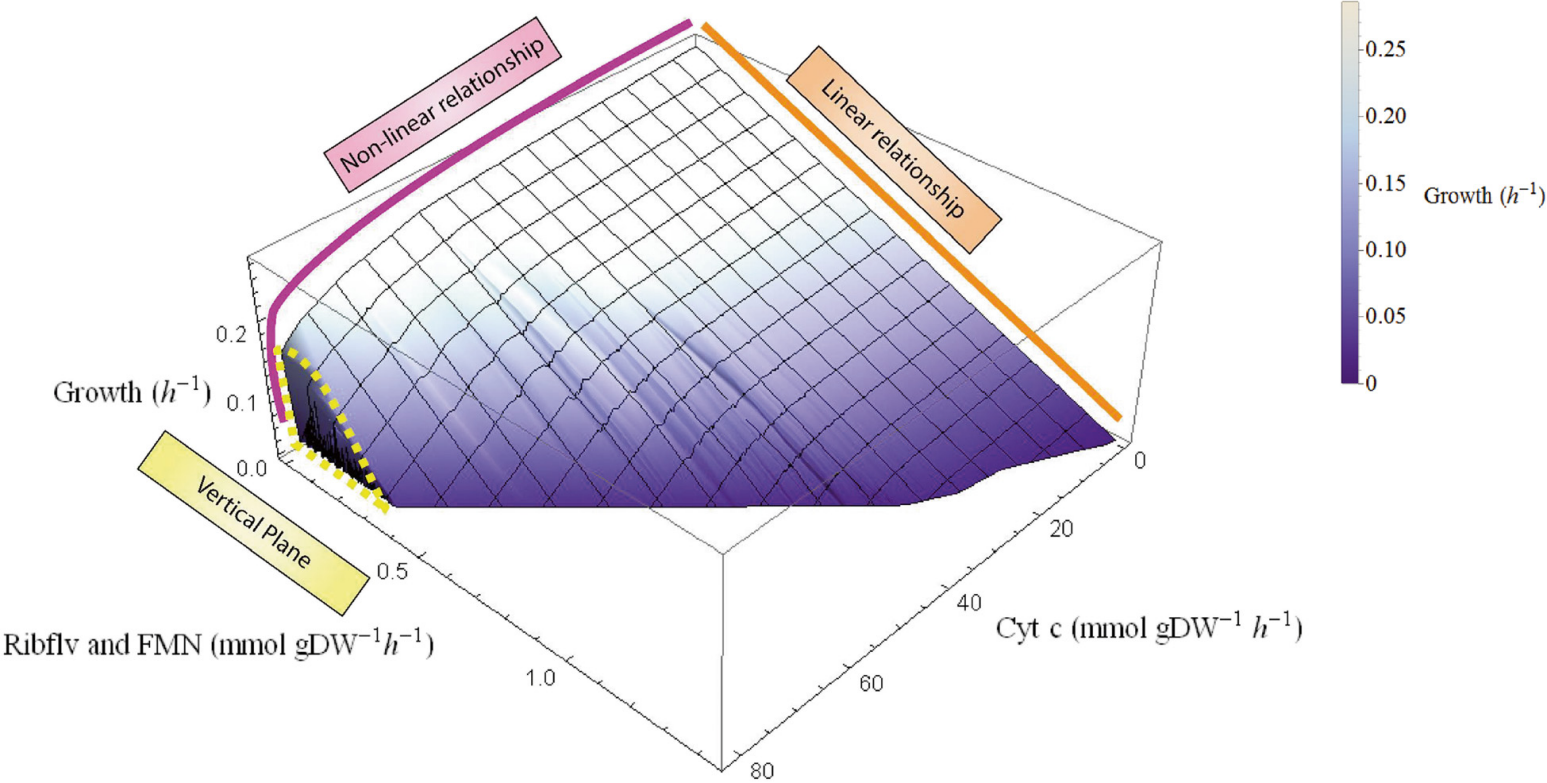
**The relationships between the flavins, cytochrome c and growth rates.** Ribflv, riboflavin; FMN, flavin mononucleotide; Cyt c, the reduced form of cytochrome c.

The three-dimensional graph (Figure 4) shows the relationship of the growth rate to the other two desired products, flavin and cytochrome c. When each of cytochrome c and riboflavin is produced alone, the biomass growth rate is non-linearly related to the cytochrome c production, but linearly proportional to the flavin production (as indicated by the pink and orange boxes in the graph). Nevertheless, if the flavins and cytochrome c are produced at the same time to sustain an energy extraction, this energy drain can cause less negative effects on the growth rates than when each electron carrier is solely produced.

The vertical plane of the graph indicates that, at lower biomass production rates (0 to 0.15 h^-1^), an increase in the flavin production rate did not always necessitate a decrease in cytochrome c production rate. When the flavin production rate was below about 0.45 mmol/gDW/h and the cytochrome c production rate was above about 78 mmol/gDW, the decrease in biomass growth did not further improve the production rates of cytochrome c.

Viewed from three different directions along the three axes labelled in the graph, the relationships between 1) cytochrome c and growth rate, 2) flavins and growth rate and 3) flavins and cytochrome c are further discussed and analysed in the following three sub-sections. The steepnesses of the curves are used in these discussions as indicators of the relative production efficiency. This is based on the interpretation that, for example, a steep downward curve means that a small increase in the quantity on the horizontal axis implies a large decrease of the complementary quantity on the vertical axis. As these are trade-offs consuming the same amount of metabolic resource, this implies that the vertical quantity is produced more efficiently than the horizontal one.

### Cytochrome c versus growth rate

**Figure 5:**
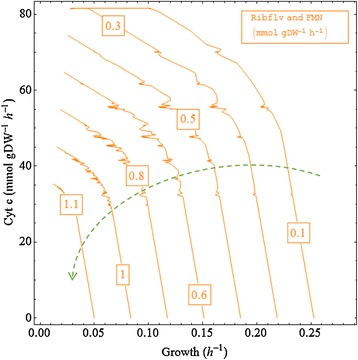
**The relationship between the cytochrome c production and biomass growth at varying secretion rates of the flavins.** Ribflv, riboflavin; FMN, flavin mononucleotide; Cyt c, the reduced form of cytochrome c.

Figure 5 shows that the increase in the synthesis rate of the flavins (indicated by the green dashed line with an arrowhead) can notably repress the maximum cytochrome c production rate from about 81 mmol/gDW/h to 35 mmol/DW/h, but hardly influences the efficiency of cytochrome c production for the cell (as evidenced by the equally spaced parallel curves). In a metabolic state, where there is no or a low flavin secretion (for example, at a synthesis rate of 0.1 mmol/gDW/h, described by the uppermost curve), the relationships between the increase in the cytochrome c production rate and the decrease in the biomass growth rate can be divided into three phases based on the production efficiency of the cytochrome c (the steepness of the curve): Phase one, a high efficiency of converting the metabolic resource into cytochrome c production when the growth rate is above around 0.23; Phase two, a low efficiency of converting the metabolic resource into cytochrome c when the growth rate is within the range of 0.17 - 0.26 h^-1^ and Phase three, a efficiency plateau which means any further decrease in the biomass growth does not improve the cytochrome c production rate. Note that as the production rate of the flavins increased (indicated by the green dashed line with an arrowhead), the curve for the cytochrome c production versus biomass growth only comprised the Phase one and two relationships, and the Phase three relationship diminished, which indicates that the flavin synthesis rate increased, with a rise in the average slope for the curves of cytochrome c versus growth rates.

The zig-zag part of the curves corresponds to flux distribution solutions switching from one metabolic state to another. These adjacent metabolic states in the zig-zag areas are randomly obtained from independent calculations, indicating that a very tiny change in the numerical value of one parameter can produce a similar but distinct metabolic state. This is thought to correspond to the biological phenomenon that cells even maintained in the same environmental condition can express individual behaviours.

### Flavins versus growth rate

**Figure 6:**
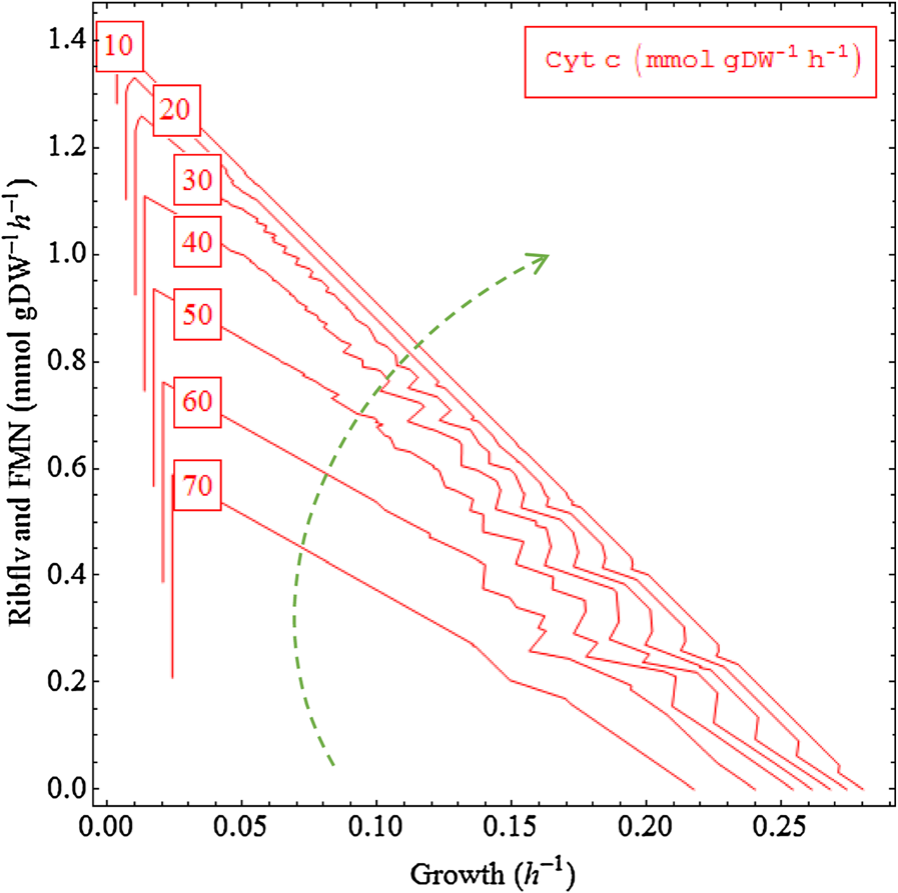
**The relationship between the secretion of flavins and biomass growth rate at varying cytochrome c production rates.** Ribflv, riboflavin; FMN, flavin mononucleotide; Cyt c, the reduced form of cytochrome c.

Figure 6 shows the plots of flavin production rates versus growth rates at different production rates of the cytochrome c. At different levels of cytochrome c production rates, in general, the flavin production rate increased with a decrease in the growth rate, until it reached an apex at the low growth rates ranging from around 0.025 to 0.04 h^-1^; it then plummeted as the growth rate approached zero. The decrease in the cytochrome c production rate not only augmented the maximum capability of the cell for flavin secretion, but it also raised the efficiency of the flavin synthesis, as is evident from the elevated slopes of the curves (indicated by the green dashed line with an arrowhead).

### Flavins versus cytochrome c

**Figure 7:**
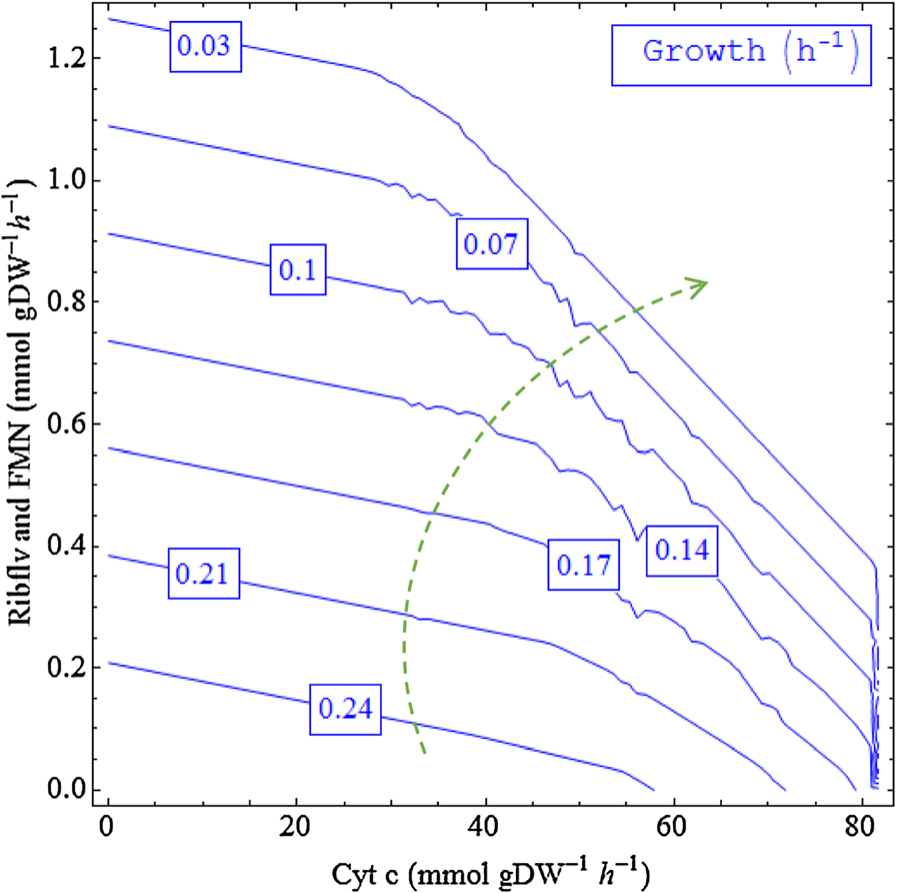
**The relationship between the secretion of flavins and production of cytochrome c at varying growth rates.** Ribflv, riboflavin; FMN, flavin mononucleotide; Cyt c, the reduced form of cytochrome c.

Figure 7 shows that the relationship between the flavins and the cytochrome c production rates changes from linear at highest growth rates to non-linear at the lower growth rates (indicated by the green dashed line with an arrowhead).The vertical axis scale (the flux range scale for the flavin production rates) is much smaller than the horizontal axis scale (the scale for cytochrome c), indicating that the metabolic cost for the synthesis of each millimole of flavins is much higher than that for production of cytochrome c.

At very high growth rates (for example, 0.24 h^-1^, corresponding to the bottom curve of the graph), there is generally a simple linear relationship between the flavin production and cytochrome c production. This indicates that, to maintain an optimal or high growth rate, all efficient metabolic pathways need to be exploited to convert metabolic resources into the biomass components, leaving no freedom for the metabolism to adjust the priority of producing flavins versus cytochrome c.

At lower growth rates (such as 0.03 h^-1^, corresponding to the top curve of the graph), based on the efficiency of conversion of riboflavin versus cytochrome c, the curves can be divided into three regions.

1. High efficiency of producing flavins compared to cytochrome c. In the metabolic states of this region (bottom right of the curve), the synthesis of cytochrome c is relatively less metabolically costly than the synthesis of flavins, and FMN did not influence the maximum capability of the cell to produce reduced cytochrome c.
2. Medium efficiency. In the metabolic states of this region (the part of the curve for which the cytochrome c production rate ranges from about 80 to 30 mmol/gDW/h), the further reduction in production rate of cytochrome c increased the flavin production with a decreased conversion efficiency (a steep slope) of metabolic resource into flavins. Compared to the previous high efficiency region, a downward curvature can be seen with decreasing slopes of the lines between the data points.
3. Low efficiency. At the left-side tail of the curve (top left of the curve), a significant drop in the cytochrome c production rate is required for a rise in the secretion rate of the flavins, which is reflected by the gentle slope of the line.

Since the microbe has a higher capability (higher upper boundary value) for cytochrome c production than for the flavins, the cytochrome c production is much more important in the metabolism. This can also be seen by the fact that the flavins change by a factor of about 5, that is, 1.44 (flavins)/0.286 (growth rate), to obtain a zero growth rate, whereas the cytochrome c changes by a much larger factor of around 286, that is, 81.68 (cytochrome c)/0.286, to reach a zero growth rate. The present modelling results indicate that a high and flexible production rate of cytochrome c is allowed by the stoichiometric constraints of the MR-1’s metabolism, which is in keeping with the experimental genome evidence of more cytochrome c-type proteins being found in the MR-1 than in other prokaryotes [32].

Our results also show that the production of riboflavins and FMN can heavily suppress the growth rate and the availability of cytochrome c. This implies that regulation of expressions of particular enzymes can cause a significant change to the metabolic flux distribution, and the metabolism of MR-1 is not as naturally optimized for cytochrome c production as for synthesis of the flavins.

### Metabolic strategies for sustaining a high flux of reducing equivalents in the three electron transfer cases

The reactions (enzymes) that were responsible for the enhanced diversion of the reducing equivalent flux towards current production are summarized in Tables 1, 2 and 3, and the metabolic mechanisms underlying the high current outputs in the three modes are discussed in the next three sections.

**Table 1:** **Identified reactions that contribute significantly to the predicted maximum cytochrome c production rate**

**Reaction ID**	**Flux**		**Enzyme**	**EC Number**	**Reaction**	**Subsystems**
	**Min**	**Max**				
CYOO2	81.68	81.68	Cytochrome-c oxidase (2 protons translocated)	1.9.3.1	(2) focytcc [c] + (4) h [c] + (0.5) o2 [c] -- > (2) ficytcc [c] + (2) h [e] + h2o [c]	Energy metabolism

(Note: the flux values are calculated by multiplying the reaction flux (not shown) and the stoichiometry coefficient of the cytochrome c.)

The flux ranges were calculated from the FATMIN algorithm.

**Table 2:** **Identified reactions that contribute significantly to the predicted maximum NADH production rate**

**Reaction ID**	**Flux**		**Enzyme**	**EC Number**	**Reaction**	**Subsystems**
	**Min**	**Max**				
GAPD	-8.745	-0.0559	Glyceraldehyde-3-phosphate dehydrogenase (NAD)	1.2.1.12	[c] : g3p + nad + pi < == > 13dpg + h + nadh	Glycolysis/Gluconeogenesis
ICDHxi	0	54.09	Isocitrate dehydrogenase (NAD)	1.1.1.41	[c] : icit + nad -- > akg + co2 + nadh	Citrate cycle (TCA)
THD5	0	54.09	NAD transhydrogenase	N/A	[c] : nad + nadph -- > nadh + nadp	Energy metabolism

(Note: The flux values are calculated by multiplying the reaction flux (not shown) and the stoichiometry coefficient of the NADH.)

The flux ranges were calculated from the FATMIN algorithm. N/A, not available.

**Table 3:** **Identified reactions that contribute significantly to the predicted maximum cytochrome c and flavin production rates**

**Reaction ID**	**Flux**		**Enzyme**	**EC Number**	**Reaction**	**Subsystems**
	**Min**	**Max**				
CYOO2	75.12	81.68	Cytochrome-c oxidase (2 protons translocated)	1.9.3.1	(2) focytcc [c] + (4) h [c] + (0.5) o2 [c] -- > (2) ficytcc [c] + (2) h [e] + h2o [c]	Energy metabolism
SO3De	0	-6.559	Sulfite dehydrogenase	1.8.2.1	(2) ficytcc [c] + h2o [e] + so3 [e] -- > (2) focytcc [c] + (2) h [e] + so4 [e]	Energy metabolism
RBFK	-0.2504	-0.2504	Riboflavin kinase	2.7.1.26	[c] : atp + ribflv -- > adp + fmn + h	Cofactor and prosthetic group biosynthesis
RBFSb	0.5008	0.5008	Riboflavin synthase	2.5.1.9	[c] : (2) dmlz -- > 4r5au + ribflv	Cofactor and prosthetic group biosynthesis
FMNRx	0.2504	0.2504	FMN reductase (NADH dependent)	1.5.1.29	[c] : fmn + h + nadh -- > fmnRD + nad	Cofactor and prosthetic group biosynthesis

(Note: Whereas the flux values for CYOO2 and SO3De are calculated by multiplying the reaction flux (not shown) and the stoichiometry coefficient of the cytochrome c, the flux values for RBFK, RBFSb and FMNRx are calculated by multiplying the reaction flux (not shown) and the stoichiometry coefficient of the riboflavin or FMN.)

The flux ranges were calculated from the FATMIN algorithm.

### DET mode

During MFC operation of the DET mode, the electrons are continuously extracted from the membrane by the anode, imposing a metabolic pressure on cellular activities. The surplus flux of the reduced cytochrome c for the DET mode was derived from the reaction CYOO2 (EC: 1.1.1.2) (Table 1). Based on the stoichiometric constraints, the metabolism of MR-1 had a potential to augment the extracellular transfer rate up to about 81.68 mmol/gDW/h for the perturbed growth.

Unlike *G. sulfurreducens*, which relied on CYOR1m (cytochrome c reductase, EC: 1.7.2.2) to regenerate reduced cytochrome c, *S. oneidensis* MR-1 did not use CYOR1m and chose CYOO2 (cytochrome c oxidase, EC: 1.9.3.1) to produce the high flux of the reduced cytochrome c.

### MET mode

As a response to draining electrons away from NADH, a metabolic mechanism that regenerates NADH from NAD^+^ at a high rate had to be activated to restore the redox balance for survival (growth). It is suggested that two mechanisms will be triggered to avoid NADH imbalance: 1) the growth rate decreases, and 2) a metabolic reconfiguration mechanism is employed by the cell to restore the balance.

In total, there were 63 reactions associated with NADH production or consumption. Among them, only two reactions, ICDHxi (EC: 1.1.1.4) and THD5, were responsible for the identified maximum net NADH flux (48.53 mmol/gDW/h) (Table 2). Each of the two reactions had a capability to solely supply up 54.09 mmol/gDW/h of NADH flux (111.5% of the maximum net value). Therefore, any possible combinations of these two reaction fluxes that made up the maximum net NADH flux were viable. The total of two reactions identified for *S. oneidensis* MR-1 was much less than the ten reactions for the MET mode of *G. sulfurreducens* [33].

Whereas ICDHxi is associated with the tricarboxylic acid (TCA) cycle, THD5 is catalysed by NADPH:NAD^+^ oxidoreductase. A high NADH flux (47.03 mmol/gDW/h) yielded from NADPH by THD5 was also observed for modelling an optimal anaerobic growth of *S. oneidensis* MR-1 in a previous study [34]. On the other hand, the possible high flux of THD5 might also be due to a lack of proper thermodynamic constraints on the reaction.

The excess fluxes (the flux portion higher than 48.53 mmol/gDW/h) were mainly consumed by GAPD (EC: 1.2.12), which can consume up to 8.745 mol/gDW/h of the NADH flux, accounting for 18.02% of the maximum net NADH flux. All other NADH-involved reactions usually have extremely low flux values (absolute flux value < 1 mmol/gDW/h) with no variability (v_i min_/v_i max_ > 0.99) under high current output. The low flux values and rigid variability reflect the fact that NADH is involved in many reactions and that increasing NADH fluxes in a few reactions can limit the variability of metabolic fluxes through many other reactions.

A comparison of the metabolic strategies employed by *S. oneidensis* MR-1 and *G. sulfurreducens* for both the DET and MET modes indicates that, although the two microbes have some similar functional features, such as nanopili and type-c cytochrome-based DET mode, the underlying metabolic mechanisms to maximize current production are different. Because, in contrast to the anaerobic *Geobacter*, the MR-1 is aerobic, the distinction in pathway selections between the two species may be ascribed to the fundamental discrepancy between aerobic and anaerobic metabolisms.

### DET mode with synthesis of riboflavin and FMN

In the mixed mode, cytochrome c and the flavins (riboflavin and FMN) were concurrently up-regulated to sustain the high reducing equivalent fluxes. As a result of maximization of the production rates of both the cytochrome c and flavins, two reactions in the metabolism, CYOO2 (EC: 1.9.3.1) and SO3De (EC: 1.8.2.1), were mainly associated with the maximum cytochrome c output flux (75.12 mmol/gDW/h) (Table 3). CYOO2 was responsible for the producing flux, contributing up to 108.7% of the maximum output flux. Any excess percentages (up to 8.7% of the maximum output) were balanced by the cytochrome c-consuming flux of the reaction SO3De (EC: 1.8.2.1). Therefore, any combination of the two reaction fluxes within the identified viable flux ranges and making up the net sum cytochrome flux of 75.12 mmol/gDW/h would be viable. The non-uniqueness of the obtained solution indicates that the secretion of the flavins improves the flexibility of the metabolism under the cytochrome c-linked DET mode.

The maximum net flux (0.5008 mmol/gDW/h) of the flavins was achieved by a combination of fluxes of three reactions, RBFK (EC: 2.7.1.26), RBFSb (EC: 2.5.1.9) and FMNRx (1.5.1.29). Half of the flux of RBFSb was responsible for the surplus riboflavin flux of 0.2504 mmol/gDW/h, and the other half was consumed to produce FMN. The riboflavin flux value was the same as the FMN flux value, indicating that the metabolic costs for synthesis of the two metabolites are the same. The three reactions underlying the flavin-linked current production had rigid flux ranges, implying that the synthesis of the flavins is limited by the diversion of metabolic resources toward biomass growth.

### Characterization of the solution space of the metabolism (a series of FATMIN solutions at varying λ)

To elucidate how the metabolism is reconfigured to cope with different levels of energy perturbation, we have examined the key solution reactions, of which the fluxes significantly contribute to a series of selected growth rates and current outputs. The results are shown in Tables 4, 5 and 6.

**Table 4:** **Summary of lists of the key reactions underlying different levels of the current output in the DET mode**

Current output (A/gDW)	0.000	0.008	0.015	0.152	0.723	1.368	2.058	2.189	2.189	2.189	2.189
Growth rate (h^-1^)	0.2864	0.2862	0.2861	0.2830	0.270	0.2553	0.1920	0.0908	0.0545	0.0272	0.0091
Reaction IDs	CYOO2	CYOO2	CYOO2	CYOO2	CYOO2	CYOO2	CYOO2	CYOO2	CYOO2	CYOO2	CYOO2
	CYOR7	CYOR7	CYOR7	CYOR7	CYOR7	CYOR7	SO3De				
	SO3De	SO3De	SO3De	SO3De	SO3De	SO3De					

(Full reaction names and detailed flux ranges calculated for each reaction can be found in Additional file 1.)

**Table 5:** **Summary of lists of the key reactions underlying different levels of the current output in the MET mode**

Current output (A/gDW)	0.0000	0.0153	0.0305	0.2861	1.0948	1.6333	2.0782	2.4347	2.5273	2.6015	2.6534
Growth rate (h^-1^)	0.2864	0.2854	0.2843	0.2669	0.2042	0.1524	0.0969	0.0505	0.0314	0.0162	0.0055
Reaction IDs	AKGD	AKGD	AKGD	AKGD	AKGD	AKGD	AKGD	GLUDx	GAPD	GAPD	GAPD
	MDH	MDH	MDH	MDH	ICDHxi	GLUDx	GLUDx	GLYCL	GLYCL	ICDHxi	ICDHxi
	PDH	PDH	PDH	PDH	MDH	ICDHxi	GLYCL	ICDHxi	ICDHxi	THD5	THD5
					NADH11	ILEDH2	ICDHxi	ILEDH2	PDH		
					NADH13	LLEUDr	ILEDH2	LLEUDr	PGCD		
					NADH16	MDH	LLEUDr	PDH	THD5		
					PDH	PDH	MDH	PGCD			
					PGCD	PGCD	PDH	THD5			
					THD5	THD5	PGCD	VALDHr			
						VALDHr	THD5				
							VALDHr				

(Full reaction names and detailed flux ranges calculated for each reaction can be found in Additional file 1.)

**Table 6:** **Summary of lists of the key reactions underlying different levels of the current output in the mixed mode**

Current output (A/gDW)	0.0617	0.0633	0.0648	0.0924	0.2135	0.3616	0.6473	1.2086	1.5694	2.0402	2.1989
Growth rate (h^-1^)	0.2011	0.2010	0.2010	0.2006	0.1986	0.1962	0.1916	0.1675	0.1328	0.0874	0.0317
Reaction IDs	CYOO2	CYOO2	CYOO2	CYOO2	CYOO2	CYOO2	CYOO2	CYOO2	CYOO2	CYOO2	CYOO2
	CYOR7	CYOR7	CYOR7	CYOR7	CYOR7	CYOR7	CYOR7	SO3De	SO3De	SO3De	RBFK
	SO3De	SO3De	SO3De	SO3De	SO3De	SO3De	SO3De	RBFK	RBFK	RBFK	RBFSb
	RBFK	RBFK	RBFK	RBFK	RBFK	RBFK	RBFK	RBFSb	RBFSb	RBFSb	FMNRx
	RBFSb	RBFSb	RBFSb	RBFSb	RBFSb	RBFSb	RBFSb	FMNRx	FMNRx	FMNRx	
	FMNRx	FMNRx	FMNRx	FMNRx	FMNRx	FMNRx	FMNRx				

(Full reaction names and detailed flux ranges calculated for each reaction can be found in the Additional file 1.)

### DET mode

As the redox perturbation increases from the lowest level (DET flux is zero) to the highest level (DET flux is maximized using a λ of 0.999926), the metabolism decreases the cytochrome c flux diverted to the two reactions, CYOR7 and SO3D3, and increases the cytochrome c production of the reaction CYOO2, to achieve a high net cytochrome c flux for the DET mode current production (Table 4).

### MET mode

A low redox perturbation of NADH_mfc drain (λ below 0.9934) did not have an impact on the choice of metabolic pathways involving NADH regeneration, when compared with the control metabolic state (Table 5). As the redox perturbation increased, more NADH regeneration pathways were activated supplying the NADH flux for the MET mode. This indicates that, at a moderate redox perturbation, the metabolism tends to use the metabolic pathways with a higher NADH regeneration efficiency, in order to leave more metabolic resources for maximization of the growth rate. However, as the redox perturbation increased to a much higher level, the reactions with higher efficiencies but lower maximum capabilities must be abandoned, so as to make the metabolic resources sufficient for higher current outputs of the MET mode. Finally, ICDHxi and THD5 are the only solutions for the extremely high NADH regeneration rate (above 48.53 mmol/gDW/h) identified previously.

Table 5 also indicates that all identified enzymes underlying the high current outputs are oxidoreductases (as indicated by the classification number EC 1; see the EC numbers of enzymes in Additional file 1). This is in keeping with the previous speculation that oxidoreductases play an important role in the reduction of external electron acceptors, such as Fe ions, under aerobic conditions [24].

### DET mode with synthesis of riboflavin and FMN

The maximum flux of the flavin secretion was relatively low (about 1.441 mmol/gDW), and to achieve such a flavin secretion rate, the cell needs to heavily decrease the reduced cytochrome c production and the growth rates (Table 6). Since the synthesis of the flavins is much more metabolically costly than the production of the reduced cytochrome c, it is not worth assigning a higher priority for the flavin production than the cytochrome c production, in the context of the overall objective of maximization of current output. Therefore, we used a lower priority (a λ of 0.023399) for optimizing the flavin secretion rate, because any higher λ values would significantly degenerate the outcomes of the other two objectives: 1) to achieve a high current output, and 2) to maintain a viable growth rate.

The simulation results obtained by varying λ for the cytochrome c output while fixing a λ of 0.023399 for the flavin secretion show that, under the mixed mode, the metabolism had rigid solutions in choosing pathways to synthesize flavins, but had flexible solutions comprising a number of different combinations of the cytochrome c-producing and consuming reactions.

### Effect of varying lactate uptake on predicted biomass and reducing equivalent production rates

**Figure 8:**
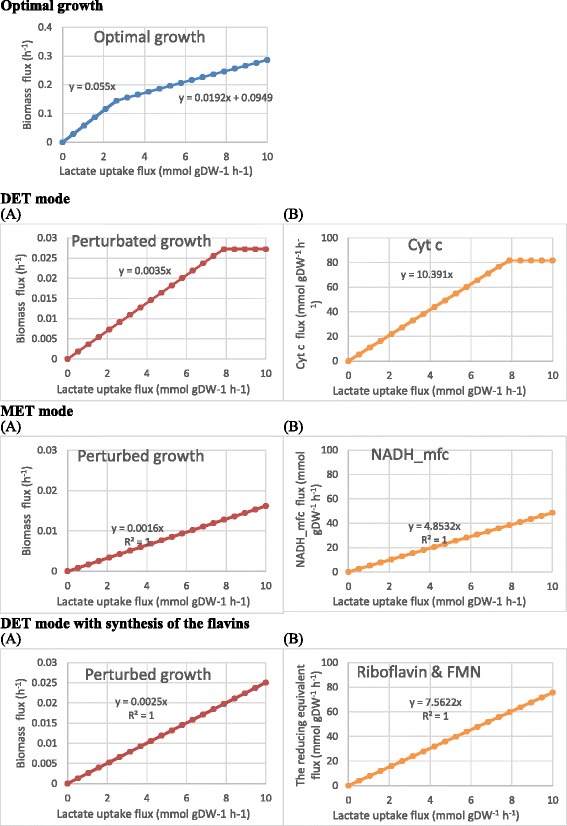
**The effect of varying lactate uptake rate on the biomass and reducing equivalent production rates in the three modes. (A)** The effect of varying lactate uptake rate on the biomass growth rates when biomass production is set as the biological priority for the organisms (use of only biomass maximization as objective function) and when the electron transfer in the three modes is set as the priority. **(B)** The effect of varying lactate uptake rate on reducing equivalent production rate. Cyt c, the reduced form of cytochrome c; NADH_mfc, the net NADH flux diverted for current production; FMN, flavin mononucleotide.

Based on the conversion efficiency of lactate to biomass, the relationship between biomass production rates and the lactate uptake rates can be categorized into two stages (Figure 8): 1) a high efficiency when the lactate uptake rate is below about 2.6 mmol/gDW/h; 2) a low efficiency when the lactate uptake rate is above 2.6 mmol/gDW/h. This is attributed to different metabolic pathways involved for different levels of growth rates.

In the DET mode, it was found that, when the lactate uptake rate was below 8 mmol/gDW, the biomass production rates or the electron transfer rates were linearly proportional to the lactate uptake rate in the MET and the mixed modes (Figure 8). However, a further increase in the lactate uptake rate did not increase the upper bounds of the cell on biomass and cytochrome c production. This suggests that lactate uptake rate is the major limiting factor for both biomass and current yields. In addition, since the Y axis scales for the biomass productions (left side, 0 - 0.03 g/h) are much smaller than those for current outputs (right side, 0 up to 100 mmol/gDW/h), the biomass production is much more lactate-costly than the current production.

The DET mode (slope = 10.391) was higher than the mixed mode (slope = 7.5622) and the MET mode (slope = 4.8532) (Figure 8). This suggests that the current output of the DET mode was inherently favoured by the metabolism since, for each ampere output, it requires much less metabolic resource than the other two modes. The lower conversion efficiency achieved by the mixed mode and the MET mode indicates that the synthesis of flavins (riboflavin and FMN) and NADH was not as metabolically efficient as the production of cytochrome c. The DET mode had a higher efficiency in energy outputs than the NAD^+^/NADH, which is the universal redox coupler in living cells for energy metabolism.

To test whether a further increase in lactate uptake rate (>10 mmol/gDW) can lead to a plateau of the flavin production, we artificially increased the literature value of lactate uptake rate, 10 mmol/gDW/h, to 15 mmol/gDW/h, and found that any lactate uptake rates higher than 11.05 mmol/gDW/h could not further increase the total redox flux production rate; the highest total redox flux production rate stayed at 82.22 mmol/gDW/h. A lactate uptake flux of 15 mmol/gDW/h may not be practical, but using such a high value in the simulation can predict the trend of the curve (that is, the conversion efficiency) for the flavin production rate versus lactate uptake rate if the innate burden on the lactate uptake capability were to be removed.

The mixed mode (slope = 7.5622) achieved a higher efficiency of converting the substrate into electricity in MFCs compared to the MET mode (slope = 4.8532), but a lower efficiency than the DET mode. This suggests that the stoichiometry of the metabolism is naturally optimized for synthesis of the reducing equivalent in the following order: cytochrome c > flavins > NADH.

Finally, the three operation modes are compared for their theoretically maximum current output (see Table 7) in the following section.

**Table 7:** **Comparison of predicted amperages and power outputs of the three modes**

	**Conditions**	**Biomass growth rate (h** ^**-1)**^	**Electron (mmol gDW** ^**-1**^ **h** ^**-1**^ **)**	**Amperage (A gDW** ^**-1**^ **)**	**Coulombic efficiency (CE%)**	**Theoretical limit of the power output (W gDW** ^**-1**^ **)**
MET	5% of maximum growth rate	0.01432	97.40	2.610	81.17%	2.166
DET			81.68	2.189	68.07%	0.5604
DET with flavin secretion			81.98	2.197	68.31%	0.5738

Note: Cytochrome c and NADH have different standard potentials. The power density for the mixed mode was calculated by summing the power contributions from the riboflavin and FMN-involved DET and the cytochrome c-dependent DET; that is, the current outputs of DETs were multiplied by their appropriate voltages, respectively.

### Comparison of amperage output of the three operation modes

The reference metabolic states for elucidating the inherent capability of *S. oneidensis* MR-1 for current output and coulombic efficiency were based on the assumption that 5% optimal growth would be the minimum viable growth rates in practice. A comparison of the results (Table 7) shows that the NADH-linked MET mode (2.610 A/gDW and 81.17% CE) can achieve a slightly higher current output and higher coulombic efficiency than the cytochrome c-based DET mode with flavin secretion (2.197 A/gDW and 68.31% CE) and the pure DET mode (2.189A/gDW and 68.07% CE). The higher coulombic efficiency values could be attributed to the fact that NADH is the redox currency used to enable many reactions including the electron transfer chain in the membrane of the prokaryote, which is the primary source sustaining the DET mode current production.

Due to the lower formal potential of c-type cytochrome and flavins, the power outputs of the cytochrome c- and flavin-dependent current generation were much lower than the NADH-dependent counterpart. This emphasizes the fact that NADH is naturally optimized as the bellwether of the electron delivery, and the maximum power output of the microbe would only be achieved if an efficient redox mediator were developed. Nevertheless, the lack of ideal mediators that can efficiently and safely extract the electrons from metabolism hinder the current development of the MET mode in reality.

*Shewanella* and *Geobacter* are both prokaryotes able to transfer the electron to the anode through nanopili-mediated DET mode, and thus, there has been a controversial question about which one of those two would be the better candidate for MFC electricity generation. Previously, we have examined the maximum potential of *G. sulfurreducens* for MFC electricity generation [33]. The comparison of the two microbes shows that *G. sulfurreducens* has a higher capability for the DET mode current production, at 3.710 A/gDW with a coulombic efficiency of 96.12%. However, it should be noted that the higher current output of *G. sulfurreducens* was achieved by a high acetate uptake rate (18 mmol/gDW/h) supplying an electron uptake flux of 144 mmol/gDW/h, which is higher than the lactate uptake rate of 10 mmol/gDW/h comprising an electron uptake flux of 120 mmol/gDW/h used in the simulation of *S. oneidensis* MR-1.

Our calculations showing that the MR-1 produced a slightly lower maximum current output than its counterpart, *G. sulfurreducens*, are generally in agreement with previous experimental opinions that *Geobacter* can generate higher current densities in MFCs than all other pure cultures [35].This conclusion was further emphasized by another observation that *G. sulfurreducens* PCA produced ten times higher current levels in lactate-fed microbial electrolysis cells than *S. oneidensis* MR-1 [36]. Despite having a lower current output potential, the unique feature of *S. oneidensis* MR-1 to secrete flavins can preclude the need for direct contact with the electrode, establishing a long-distance electron transfer without involvement of exogenous mediators and can be exploited in the development of some particular applications.

Compared with the sole DET mode, the present calculation did not show a further increase in the total current output for the assumed DET mode with flavin secretion, and any flavin synthesis rate higher than 0.2587 mmol/gDW/h can significantly reduce the overall capability of the cell for current generation. This is partially contradictory to a previous conclusion that the two electron-mediated mechanisms - the nanowires-mediated electron transport and secretion of flavins - together determine the efficiency of the current generation in *Shewanella* containing MFCs [37].

Previously, the flavins (riboflavin and FMN) were found to contribute as high as 73% of the total current generated in an MFC using *S. oneidensis* [18]. Therefore, to enhance the electricity output of MFCs, there have been research efforts that tried to improve the flavin biosynthesis in *Shewanella* [38]. By contrast, our results indicate that the synthesis of the flavins can reduce the capability of the organism for cytochrome c-based DET and growth rates, and should be avoided to achieve a high overall current output. Our model assimilates the whole genome-scale metabolism of the *Shewanella*, and focuses on elucidation of the overall capability of a single cell for extracellular electron transfer. This is different from previous MFC studies that used to report the current output for the whole cell culture in MFCs, which resulted from a combination of contributions from different electron transfer mechanisms of cellular phenotypes, namely, secreted flavins of cells distant from the electrode and cytochrome c of cells near the electrode. Therefore, the observed importance of flavin secretion for cell culture-based extracellular electron transfer should not decrease the priority to develop cytochrome c-based DET mode for high current outputs in future studies.

The MET mode of *G. sulfurreducens* (2.771 A/gDW) achieved a slightly higher maximum current output than that of *S. oneidensis* MR-1 (2.610 A/gDW), but with a lower CE of 71.79%. The use of exogenous electron shuttles in the MET mode of an MFC may be toxic to the normal metabolism of the MR-1, but can improve the current output when the self-synthesized flavins are not sufficient to convey all the available electrons inside the metabolism towards the electrode. This was confirmed by the observation that the addition of exogenous soluble flavins into the media can improve the electron transfer rates of an MFC [39].

Experimentally, it is very difficult to measure the number of cells that actually contribute to the observed electric current output. Consequently, the MFC experimental studies often report the current density as current generated per unit area of the anode surface area (A/cm^2^) or current generated per unit volume of the cell (A/m^3^). Since different MFC studies use different configurations involving different types of reactors, surface area and types of electrodes (for example, mesh, plate or multi-layer), it is difficult to directly compare current output in these units (A/m^2^ or A/m^3^) with the unit (A/gDW) in the metabolic flux model. The advantage of the flux unit (A/gDW) is that it allows the calculated current density values to be applied to any MFC configuration by taking into account the cells that are actually contributing to the current generation in that situation.

A recent study has reported a maximum current output of 11.09 ± 0.28 A/m^2^ for an MR-1-based MFC, and a total biomass of the anode biofilm of 200.7 ± 2.8 mg (fresh weight)/cm^2^ [40]. With these two values, we can translate the reported values into an estimated current output in the units of amperes per gram, 0.005525 A/g (fresh weight). This value is much lower than our calculated value, 2.197 A/gDW, for the DET mode with flavin secretion. Such a large discrepancy may be attributed to the fact that 1) the current density should be higher based on the dry weight than the fresh weight, which includes a large portion of water; 2) not all the cells in the biofilm contribute to the current output; and 3) the flavin-based current output makes up a large percentage of the observed current production, which suppresses the overall current output. On the other hand, from a positive point of view, the big discrepancy also indicates that there is still potential to exploit MR-1 to obtain a higher current output.

Based on the previous study, *S. oneidensis* MR-1 has an unusually high growth rate-dependent ATP requirement (GAR) [34]. Such a value is chosen in order to reflect the observed high consumption rate of energy content in the biomass formation. In the present modelling, since the biomass equation of the *Shewanella* model contains a high stoichiometric value for the ATP, the free electron fluxes divertible for electricity generation are suppressed. Although the high GAR is required to account for the fact that energy-dissipating futile cycles can be activated in *S. oneidensis* MR-1 under aerobic conditions [34], in the wild environment, the microorganisms are more likely to experience suboxic and anoxic conditions than high O_2_ concentrations or O_2_-rich conditions, which may lead to a lower GAR, leaving more free electrons for diversion towards the electrode.

Furthermore, because the current metabolic network cannot distinguish the difference in the metabolic cost and pathways underlying the production of the two flavin molecules, riboflavin and FMN, the priorities for these two flavin molecules are set to be the same in the modelling. However, the phenomenon that the two flavins are exchangeable based on the stoichiometric constraints of the model may be attributed to the incomplete set of constraints in the model (such as enzyme capacity, regulatory, thermodynamic, or other constraints). To further investigate the relationships between syntheses of riboflavin and FMN, integration of the thermodynamic constraints into the metabolic model is required, to quantitatively differentiate the metabolic costs of the syntheses of the two redox molecules. In addition, identification of the kinetic parameters through isotopic tracers such as 13C-metabolic flux analysis and measurement of enzymatic concentrations can also elucidate the exact relationships between riboflavin and FMN for a particular metabolic state of the cell.

## Conclusions

In this study, we integrated electron transfer mechanisms with a genome-scale metabolic mode to determine the capability of *S. oneidensis* MR-1 for MFC current production and examine the properties of alternative genotypes arising from the energy perturbation. The results show that *S. oneidensis* MR-1 has the potential to produce an electric current at up to 2.610 A/gDW in the DET mode, 2.189 A/gDW in the MET mode and 2.197 A/gDW in a presumed mixed mode based on cytochrome c and flavins.

To maximize the current output, the DET mode relied on only one reaction, mediated by cytochrome c oxidase (EC: 1.9.3.1), and the NADH-linked MET mode was mainly contributed by two reactions, catalysed by isocitrate dehydrogenase (NAD) (EC: 1.1.1.41) and NAD transhydrogenase. The maximum secretion rate of flavins was quite low, namely, 1.414 mmol/gDW/h, and the flavin production can lead to significantly repressed growth and c-type cytochrome production rates. Therefore, although the flavin secretion can improve the long-distance electron transfer in the absence of exogenous mediators, it can significantly curtail the objective to maximize the overall availability of the electrons for the electrode.

Furthermore, the comparison of *G. sulfurreducens* and *S. oneidensis* MR-1 shows that the two microbes are likely to employ different mechanisms to sustain the high current outputs. This suggests that the differences in the fundamental metabolic mechanisms, in addition to the engineering aspects of the configuration, should be taken into account in the optimization of the performance of MFCs based on different organisms.

## Methods

### Modelling assumptions

The modelling conducted was based on seven assumed conditions: i) Only a pure *S. oneidensis* MR-1 culture is used for electricity generation. ii) The MFC reactor is fed continuously, providing all the necessary nutrients to meet the requirements for optimal microbial growth. iii) Sufficient oxygen was present to ensure the aerobic metabolism of *S. oneidensis* MR-1. Oxygen intrusion into MFCs used to be considered detrimental since it can intercept the electrons from anodes, resulting in a lower coulombic efficiency. However, recent studies have suggested that addition of oxygen to the anodic chamber does not necessarily lower the performance of MFCs based on the pure culture *S. oneidensis* MR-1, and instead can prominently increase their maximum current outputs [29–31]. The aerobic metabolism can lead not only to a higher biomass formation rate, but also to an enhanced electron flux to the electrode [30]. iv) In the DET mode, c-type cytochrome is the intracellular electron donor. v) In the flavin-mediated electron transfer, flavin derivatives, that is, riboflavin and FMN, are the sources for the electric circuit. vi) In the NADH-based MET mode, a putative ideal mediator (such as Neutral red [41]) can be found to enter the cytoplasm, intercept the electrons from NADH-involved metabolic activity and convey the electrons to the anode. vii) The uptake rate of the organic substrate (lactate) is allowed to vary but is constrained to a realistic range to reflect the observed uptake rates for optimal growth.

**Figure 9:**
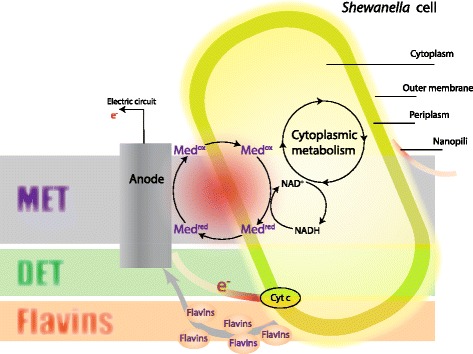
**A schematic of the electron transfer routes of** ***Shewanella oneidensis*** **MR-1.** (i) MET, the electron transfer driven by a mediator-used process; red, reduced form; ox, oxidized form. (ii) DET, the electron transfer involving c-type cytochrome. (iii) Flavins, the electron transfer depends on the self-secreted flavins conveying electrons inside the metabolism to the anode. Microbes take up substrate generating carbon dioxide and protons. This process yields electrons for metabolic benefit, that is, growth, and reduces Med_ox_ in the cytosol into Med_red_. Med_red_ diffuses into contact with the electrode, where Med_red_ reduces the electrode, generating electrical current. The oxidized form, Med_ox_, diffuses back through the anolyte for reuse by the microbes. Periplasm only exists in Gram-negative bacteria such as *S. oneidensis* MR-1.

The interactions with an MFC anode were captured by adding additional transport reactions into the model. These reactions are subject to the mass balance law in the FBA modelling. The process of conveying electrons towards the electrode is schematically shown in Figure 9.

### Multi-objective optimization

The objective equation was formulated as described in previous studies [42]. Briefly, a multi-objective equation comprises: 1) one objective that represents the maximization of the growth rate, which reflects the belief that microbes evolve to optimize their metabolisms for maximizing biomass production (growth rate) [43–46] and has been experimentally validated by numerous studies [44,47,48], and 2) other objectives that represent the maximization of the production of an overflow flux of desirable reducing equivalents such as cytochrome c, FMN, riboflavin and cytoplasmic NADH, in order to restore the redox balance perturbed by the electron extraction process during MFC operation. The multi-objective optimization involves creation of a Pareto-optimal space to optimize competing objectives in the modelling [49], forming a Pareto front that is considered the best solution that satisfies the conflicting objectives. By varying the relative weights associated with each objective [50], the impact of the enhanced energy extraction on the growth of the cell can be evaluated. The multi-objective equation in this study is defined in terms of the biomass growth flux *F*_*B*_ and the reducing equivalent diversion flux *F*_*N*_. There are two objective formulations; one (Equation 1) is for modelling the DET and MET modes, the other (Equation 2) is for the DET mode with the flavin secretion.

1 $$ o\kern0.5em =\kern0.5em \left(1\kern0.5em -\kern0.5em \lambda \right){F}_B\kern0.5em +\kern0.5em \lambda {g}_N{F}_N $$

Here ${F}_B\;\mathrm{and}\;{F}_{N_0}$ denote the biomass growth flux and reducing equivalent diversion flux (namely, the net reduced cytochrome c production flux in the DET mode or the net NADH flux in the MET mode). In the model, *F*_*B*_ and *F*_*N*_ are measured respectively in units of mmol/gDW/h and g/gDW/h or (h^−1^). For the MET mode, *g*_*N*_ is set as 0.66343 g/mmol, which is the molar mass of NADH, and for the DET mode, *g*_*N*_ is set as 0.8869 g/mmol, which is the molar mass of cytochrome c. These *g* values ensure that the λ is a dimensionless fraction that can be directly interpreted as the relative contribution of the reducing equivalent fluxes to the combined objective.

2 $$ o\kern0.5em =\kern0.5em \left(1\kern0.5em -\kern0.5em {\lambda}_b\right)\left[\left(1\kern0.5em -\kern0.5em {\lambda}_a\right){F}_B\kern0.5em +\kern0.5em {\lambda}_a{g}_{N_0}{F}_{N_0}\right]\kern0.5em +\kern0.5em {\lambda}_b{\displaystyle {\sum}_{i=1,2}\kern0.5em {g}_{N_i}{F}_{N_i}} $$

Here ${F}_B,{F}_{N_0},{F}_{N_1}\kern0.5em \mathrm{and}\kern0.5em {F}_{N_2}$ denote the biomass growth, cytochrome c production, and riboflavin and FMN fluxes, respectively. ${g}_{N_0},{g}_{N_1}\kern0.5em \mathrm{and}\kern0.5em {g}_{N_2}$ are set as 0.8869, 0.4563 and 0.3763 g/mmol, respectively. These values are molar masses for cytochrome c, FMN and riboflavin. In contrast to Equation 1, where only one λ is used, Equation 2 involves two different λ’s: λ_a_ and λ_b_. The one (λ_a_) inside the square brackets adjusts the priority between biomass and cytochrome c, and then the other (λ_b_) adjusts the priorities between the flavins and the combination of the biomass and cytochrome c.

### Fractional benefit analysis

A fractional benefit analysis, as described previously [51,52], was conducted to quantify how each of the multiple objective terms will contribute (positively or negatively) to the overall benefit. In the analysis, a value of the quantity, designated as the fractional benefit *B*, is computed by adding up the fractions of the maximal values of the objectives that they can ever achieve in a particular metabolic state. Equation 3 is used for the calculation of *B* values for a dual objective optimization (for example, MET and DET modes), whereas Equation 4 is used for a triple objective optimization (for example, DET with flavin secretion).

3 $$ B\kern0.5em =\kern0.5em \frac{1}{2}\left(\frac{F_B}{F_{\overset{*}{B}}}\kern0.5em +\kern0.5em \frac{F_N}{F_{\overset{*}{N}}}\right) $$

4 $$ B\kern0.5em =\kern0.5em \frac{1}{2}\left(\frac{1}{2}\left(\frac{F_B}{F_{\overset{*}{B}}}\kern0.5em +\kern0.5em \frac{F_{N_0}}{F_{{\overset{*}{N}}_0}}\right)\kern0.5em +\kern0.5em \frac{1}{2}\left(\frac{F_{N_1}}{F_{{\overset{*}{N}}_1}}\kern0.5em +\kern0.5em \frac{F_{N_2}}{F_{{\overset{*}{N}}_2}}\right)\right) $$

Here ${F}_B,{F}_N,{F}_{N_0},{F}_{N_1}\kern0.5em \mathrm{and}\kern0.5em {F}_{N_2}$ are the flux values obtained from FBA modelling; the maximal values ${F}_{\overset{*}{B}},{F}_{\overset{*}{N}},{F}_{{\overset{*}{N}}_0},{F}_{{\overset{*}{N}}_1}\kern0.5em \mathrm{and}\kern0.5em {F}_{{\overset{*}{N}}_2}$ are obtained when each objective is independently maximized. A maximum *B* value of 100% would be attained if a metabolic state reaches the “utopian” ideal of simultaneously producing the maximal growth rate and the maximal reducing equivalent production. On the other hand, based on Equation 3, the wild-type phenotype of maximal growth rate and no reducing equivalent has a *B* value of 50%. *B* values above 50% signify that the gain in achieving the target objective (that is, maximization of the metabolite of interest in the analysis) outweighs the losses in the others, and vice versa for values below 50%.

Based on Equation 4, the DET mode accounts for a maximum base *B* value of 50% (25% for biomass formation and 25% for cytochrome c production), whereas the two flavins (riboflavin and FMN) combined give a base *B* value of 50% (with each of the two producing a base value of 25%). A value of 25% indicates that either a wild phenotype of maximal growth rate or a maixmum cytochrome c production rate is achieved. In the theoretically ideal situation, a maximum *B* value of 100% would be reached, implying that the flavin secretion is not metabolically competitive with the biomass and cytochrome c productions. In reality, the productions of the various metabolites often involve different combinations of pathways and thus compete with one another for the limited pools of cellular resources, resulting in *B* values lower than 100%. Similar to the dual objective formulation (Equation 3), a *B* value higher than 50% obtained for the flavin secretion indicates that the gain in achieving the target objective outweighs the loss in the other two objectives (biomass growth and cytochrome c production), and vice versa for values below 50%.

This *B* measure was used in Figure 2 to highlight the way that different growth conditions produce different responses in terms of combining cell growth and external current yield.

### Calculation of output parameters

Current (in amperes) was integrated over time and converted to electrons recovered by using the following conversions: 1 C = 1 A × 1 s, 1 C = 6.24 × 10^18^ electrons, and 1 mol = 6.02 × 10^23^ electrons (Faraday’s constant is 96,485 C/mol). Therefore, one flux unit (mmol/g/h) can be converted into A/g as follows:

$$ 1\;\mathrm{mmol}/\mathrm{g}/\mathrm{h}=\frac{1\kern0.5em \mathrm{mol}}{1,000\mathrm{g}\kern0.5em \times \kern0.5em 3,600\mathrm{s}}\kern0.5em \times \kern0.5em \frac{96,485\mathrm{C}}{\mathrm{mol}}=0.0268\mathrm{A}/\mathrm{g} $$

Coulombic efficiency (CE) is commonly used to quantify the performance of MFCs and is defined as the ratio of electrons transferred to the anode to that in the starting substrate. We use the full oxidation of lactate with oxygen as the oxidant as the reference reaction to characterize the energy efficiency of the respiratory metabolism:

Lactate oxidation reaction: C_3_H_6_O_3_ + 3 H_2_O → 3 CO_2_ + 12 H^+^ + 12 e^-^.

Each molecule of lactate oxidation can donate up to a maximum of 12 electrons per molecule if all of the carbon is oxidized to CO_2_, such as in the aerobic conditions [30,53].

$$ \mathrm{CE}\%\kern0.5em =\kern0.5em \frac{{\mathrm{C}}_{\mathrm{output}}}{{\mathrm{C}}_{\mathrm{subtrate}}}\kern0.5em \times \kern0.5em 100\%\kern0.5em =\kern0.5em \frac{\mathrm{The}\ \mathrm{electron}\ \mathrm{flux}\left(\mathrm{mmol}/\mathrm{gDW}/\mathrm{h}\right)\kern0.5em \times \kern0.5em 100}{\mathrm{lactate}\ \mathrm{uptake}\ \mathrm{rate}\left(\mathrm{mmol}/\mathrm{gDW}/\mathrm{h}\right)\kern0.5em \times \kern0.5em 12}\% $$

An upper limit for the cell voltage is calculated in this work based on formal potentials of the biological and electrochemical redox processes, as given by:

$$ \Delta {E}_{\mathrm{cell}}^{o\prime}\kern0.5em =\kern0.5em {E}_{\mathrm{cathode}}^{o\prime}\kern0.5em -\kern0.5em {E}_{\mathrm{anode}}^{o\prime } $$

Here, $\Delta {E}_{\mathrm{cell}}^{o\prime }$ is the standard cell potential (that is, the electromotive force); ${E}_{\mathrm{cathode}}^{o\prime }$ is the standard potential of cathode oxidation; ${E}_{\mathrm{anode}}^{o\prime }$ is the standard potential of anode reduction. The formal potentials of the anode and cathode used for calculation of power density in the three operation modes are summarized in Table 8. In practice, the actual potential derived from the MFC will be lower due to a number of factors, including ohmic resistances, concentration polarization and kinetic constraints [54], which are not considered in the present calculations. The MFC standard cell potential calculated as below (see Table 9) indicates that as long as the same electron donors are used, the choice of biocatalysts will have little effect on the cell potential.

**Table 8:** **The standard potential of the redox reactions involving the electron donor and electron acceptor for the MFC (measured at pH 7)**

		**Redox couple**	***E°*** **′ (V)**
Anode	DET	Cytochrome c (Fe^3+^) + e^−^ → Cytochrome c (Fe^2+^)	+0.254 [55]
	DET (FMN)	FMN + 2 H^+^ + 2e → FMNH_2_	-0.216 [56]
	DET (riboflavins)	RBF + 2 H^+^ + 2e → RBFH_2_	-0.208 [56]
	MET	NAD^+^+ H^+^+2e^−^ → NADH	-0.320 [55]
Cathode		O_2_ + 4H^+^+4e^−^ → 2H_2_O	+0.51 [6,57,58]

**Table 9:** **The theoretical limit of standard anode potentials of MFC based on** ***S. oneidensis*** **MR-1**

**Electron transfer mode**	${\mathbf{E}}_{\mathbf{anode}}^{\mathbf{o} \prime }$	${\mathbf{E}}_{\mathbf{cathode}}^{\mathbf{o} \prime }$	$\varDelta {\mathbf{E}}_{\mathbf{cell}}^{\mathbf{o} \prime }$
MET	-0.32	0.51	0.83
DET (cytochrome c)	0.254	0.51	0.256
FMN	-0.216	0.51	0.726
Riboflavins	-0.208	0.51	0.718

### Simulating *Shewanella* growth

The iSO783 metabolic construction of *S. oneidensis* MR-1 was chosen as the analysis backbone for all the simulations [34]. This metabolic network has previously been quantitatively verified for aerobic growth [34]. *S. oneidensis* uses lactate as the sole source of carbon and energy. When lactate is sufficient, the *Shewanella* strain produces pyruvate and acetate, and the carbon utilization is in the following order: lactate ➔ pyruvate ➔ acetate [59]. In the present work, the maximum uptake rate of the carbon source (lactate) was set to be 10 mmol/gDW/h [34]. The maximum secretion rates of pyruvate and acetate were set to 0.872 and 3.134 mmol/gDW/h, respectively, as previously done [60]. These values were chosen to simulate the optimal growth of the *Shewanella* strain. The maximum oxygen consumption rate was constrained to -20.42 mmol/gDW/h [34]. The oxygen is the only electron acceptor; the fumarate uptake rate was constrained to zero, since it was considered as an electron acceptor for the cell to grow under anaerobic conditions [34]. CO_2_, Fe^2+^ Fe^3+^ Na^+^, NH_4_^+^, PO_4_^3-^, NO_2_^-^, NO_3_^-^ and SO_4_^2-^ were allowed to freely enter and leave the network, since they are required for optimal growth of the cell, as described previously [61]. The complete list of exchange constraints used in the study can be found in Additional file 1.

Setting a lower limit on growth rate is necessary to ensure that the cell maintains a viable metabolic state at which the redox molecules (for example, cytochrome c and flavins) can be derived from the metabolism. To obtain a realistic upper value for the current that can be extracted from a microbe, 5% of the maximum theoretical biomass production rate was chosen as the viability threshold for computational identification of the lowest growth rate.

The flux balance analysis (FBA) and flux variability analysis with target flux minimization (FATMIN) were implemented in MATLAB (The MathWorks Inc., Natick, MA), using the ORCA toolbox [62]. The maximum reducing equivalent production rates for arbitrary growth rates between the data points were calculated using the interpolation function in Mathematica 9.0 (Wolfram Research, Inc. Champaign, IL, USA).

## Additional file

Additional file 1:
**1) FATMIN results for the DET mode; 2) FATMIN results for the MET mode; 3) FATMIN results for the DET with flavin secretion mode; 4) exchange constraints; 5) the list of reaction abbreviations and full names.**

## Abbreviations

CE: coulombic efficiency
Cyt c: reduced cytochrome c
DET: direct electron transfer
FATMIN: flux variability analysis with target flux minimization
FBA: flux balance analysis
FMN: flavin mononucleotide
FVA: flux variability analysis
MET: mediated electron transfer
MFC: microbial fuel cell
NAD: nicotinamide adenine dinucleotide
NADH: reduced nicotinamide adenine dinucleotide
NADH_mfc: the net NADH flux diverted for current production
